# Clinical and prognostic implications of pituitary tumor-transforming gene 1 in solid tumors

**DOI:** 10.3389/fonc.2026.1734781

**Published:** 2026-06-26

**Authors:** Peng Dong, Weiguang Zhou, Longhui Xiong, Zhijian Li, Zhanqiao Zhang, Jianyang Yang, Huan Cao

**Affiliations:** 1 Department of General Surgery, the Second Hospital & Clinical Medical School, Lanzhou University, Lanzhou, China; 2 Department of Hepatobiliary Surgery, Shenzhen Bao ‘an District People’s Hospital, Shenzhen, Guangdong, China

**Keywords:** cancer, meta-analysis, prognosis, PTTG1, survival outcomes

## Abstract

**Background:**

Pituitary tumor-transforming 1 (PTTG1) plays a crucial role in cancer pathogenesis and has been associated with survival outcomes and clinicopathological features in patients with various tumors. Therefore, we conducted a comprehensive meta-analysis to evaluate the prognostic and clinicopathological significance of PTTG1 expression in solid tumors.

**Methods:**

A comprehensive search of the PubMed, EMBASE and Web of Science was performed to identify eligible studies until July 20, 2025. The hazard ratios (HRs) and odds ratios (ORs) with 95% confidence intervals (CIs) were calculated. Stata SE 12.0 software was used to perform the meta-analysis.

**Results:**

22 studies from 21 articles were included in this meta-analysis. The pooled results showed that patients with high PTTG1 expression had poorer overall survival (OS)(HR:1.85,95%CI:1.55-2.20) and disease-free survival/recurrence-free survival (DFS/RFS) (HR:3.48, 95%CI:1.81-6.70). Subgroup analysis showed that high PTTG1 expression was mainly associated with worse OS in renal cell carcinoma(RCC),non-small cell lung cancer(NSCLC),breast cancer(BC), esophageal squamous cell carcinoma(ESCC) and laryngeal cancer(LRGC). Furthermore, high PTTG1 expression was significantly associated with tumor stage (III-IV vs I-II), T stage (T3–4 vs T1-2), lymphatic invasion(Yes vs No) and metastasis (Yes vs No).

**Conclusion:**

PTTG1 may serve as a prognostic indicator in specific cancers.

## Introduction

In recent years, cancer remains a major global public health burden (1). Surgery, chemotherapy, and radiotherapy are widely used for cancer treatment, but these therapeutic strategies fail to provide satisfactory long-term outcomes (2). Molecular-targeted therapies have emerged as promising treatment strategies for cancer and has been proposed to be a promising strategy to largely improve long-term outcomes of cancer patients (3). Therefore, it is urgently necessary to identify effective molecular biomarkers, which can accurately predict the prognosis of patients with cancer and be developed as individualized therapeutic targets.

Human pituitary tumor-transforming gene 1 (PTTG1) is a novel oncogene, which was first identified in rat pituitary tumor cells in 1977 (4). PTTG1 is located on human chromosome 5 (5q35.1) and contains five exons and four introns. It encodes a 202-amino-acid protein, which is predominantly localized in the cytoplasm under physiological conditions (5, 6). Under physiological conditions, PTTG1 is expressed in the tissues with high proliferative activity, such as testis, thymus gland, and spleen, whereas it is rarely detected in differentiated mature tissues (7, 8). PTTG1 has been found to be aberrantly upregulated in most human malignancies and plays a crucial role in cancer pathogenesis by promoting tumor cell proliferation, epithelial-mesenchymal transition(EMT), migration, invasion, angiogenesis, and therapeutic resistance (9–11). Thus, PTTG1 is gradually emerging as a potential therapeutic target for cancer management. Increasing evidence has shown that PTTG1 overexpression is associated with poor prognosis in patients with various cancers (12–30). However, some studies report inconsistent findings (31, 32). The results of individual studies may not be reliable enough due to the limitations of methodology and sample size. Therefore, we collected the relevant literature and performed a comprehensive analysis to assess the clinical value of PTTG1 expression in different cancers.

## Methods

### Search strategy

Medical Subject Headings (MeSH) terms of PTTG1 (e.g. “PTTG1”, “pituitary tumor-transforming gene 1) in combination with cancer-related words (e.g. “cancer”, “tumor”, “carcinoma”, “neoplasm” or “malignancy”) and (e.g. “prognosis”, “survival”, “prognostic” or “outcome”) were used to retrieve eligible studies in PubMed, EMBASE and Web of Science databases until July 20 2025. Moreover, the reference lists of the included articles were also examined to identify potentially relevant studies. This meta-analysis was conducted in accordance with the Preferred Reporting Items for Systematic Reviews and Meta-Analyses (PRISMA) guidelines.The PRISMA checklist was provided as **Supplementary Materials**.

### Inclusion and exclusion criteria

Studies satisfying the following criteria were considered eligible: (1) patients were diagnosed with cancers; (2) the prognostic value of PTTG1 in cancers was reported. (3) survival outcomes were reported. (4) The article was published in English. The exclusion criteria: (1) irrelevant topics, conference abstracts, letters, review articles, comments, or animal studies; (2) survival data was incomplete; (3) studies based exclusively on public databases (e.g., TCGA, GEO) were excluded to avoid data duplication and ensure independence of patient cohorts;(4) studies involving overlapping patient cohorts.

### Data extraction and quality assessment

Two investigators independently extracted the data from included studies. Any disagreement was resolved to achieve a consensus by the third investigators. The following information was extracted from each study: first author, publication year, country, cancer type, sample size, detection method, overall survival (OS), disease-free survival/recurrence-free survival (DFS/RFS) and some clinicopathologic features including gender, T stage, distant metastasis, TNM stage, differentiation and lymphatic invasion. Multivariate analyses were preferentially selected. If some studies did not provide HRs directly, Engauge Digitizer version 4.1 was used to extract survival data from Kaplan–Meier curves (33). The quality of included studies was assessed using Newcastle-Ottawa Scale (NOS) (34). In this score system, three aspects (subject selection, comparability of subject, and clinical outcome) were evaluated and the scores ranged from 0 to 9. A score of ≥7 indicated high methodological quality.

### Statistical analysis

Stata Software 12.0 was used to perform the statistical analyses. The pooled hazard ratios (HRs) and odds ratios (ORs) with 95% confidence intervals (CIs) were calculated to assess the prognostic and clinicopathological value of PTTG1 expression. Cochran’s Q test and the Higgins I-squared statistic were conducted to explore the heterogeneity of pooled results. In cases of significant heterogeneity across studies (I2≥50% or p<0.05), a random-effects model (DerSimonian-Laird method) was chosen for the calculation of the pooled HRs or ORs. Otherwise, a fixed-effects model (the Mantel-Haenszel method) was used. Subgroup analysis was performed to explore the potential sources of heterogeneity. In addition, sensitivity analysis was performed to evaluate the stability of results. Begg’s test and Egger’s test were utilized to assess publication bias (35). If there was significant publication bias, the trim-and-fill method was adopted to further determine the reliability of the pooled result (36). P <0.05 was considered statistically significant.

## Results

### Study search

**Figure 1:**
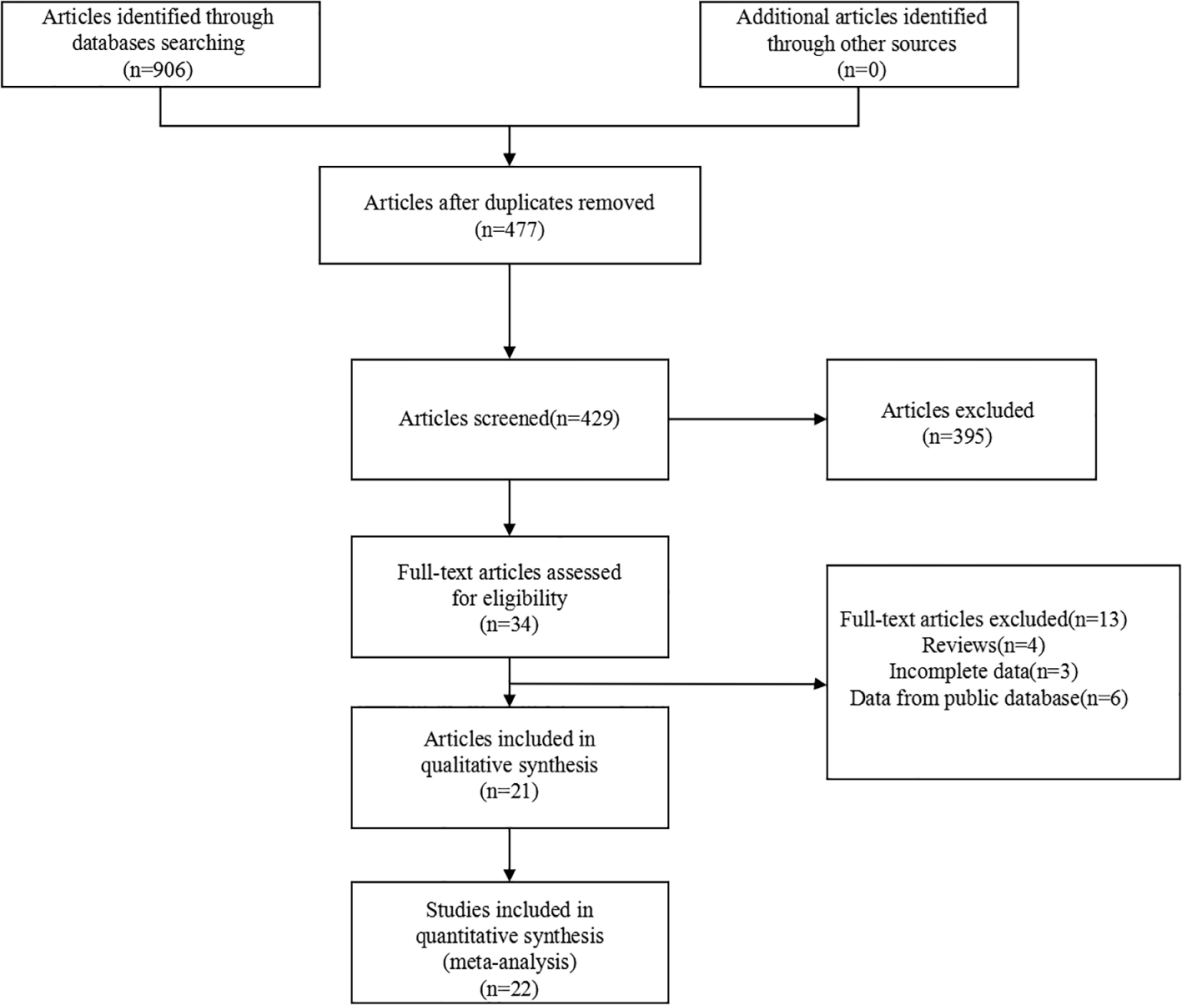
The flow chart of studies selecting process.

A total of 906 articles were retrieved from the initial database search. 477 duplicate articles were excluded. After screening titles and abstracts, we further excluded 395 for irrelevant topics, animal studies, conference abstracts and comments, with 121 articles left for full-text assessment. During full-text screening, 13 articles were further excluded. Finally, 22 studies from 21 articles were included in the final analysis (12–32). The flowchart was shown in **Figure 1**.

### Characteristics of included studies

The main characteristics of the 21 articles were shown in **Table 1**. These studies were conducted using a retrospective design and were published from 2002 to 2020. Across all included studies, the sample size ranged from 22 to 310. PTTG1 expression of 17 articles was detected at the protein level, and four articles determined PTTG1 expression at the mRNA level. Nine articles were from China, four articles from Japan, and the remaining studies were conducted in Europe and North America. A total of ten different cancer types were analyzed, including esophageal squamous cell carcinoma (ESCC), renal cell carcinoma (RCC), lung cancer (LC), hepatocellular carcinoma (HCC),laryngeal cancer (LRGC), breast cancer (BC), colorectal cancer (CRC), adrenocortical carcinoma(ACC), prostate cancer(PC) and glioma. The NOS scores of included studies ranged from 6 to 7.

**Table 1:** Basic information of included studies.

Author	Year	Country	Cancer type	Sample size	Detected method	Source of HR	Survival analysis	Analysis type	NOS score
Fujii (12)	2006	Japan	HCC	62	mRNA	Reported	OS,DFS	Multivariate	7
Genkai (13)	2016	Japan	Glioma	44	Protein	Reported	OS	Univariate	7
Ito (31)	2008	Japan	ESCC	113	Protein	Reported	OS	Multivariate	7
Li (14)	2013	China	NSCLC	146	Protein	Reported	OS	Univariate	6
Ma (15)	2018	China	LRGC	210	Protein	K-M curves	OS	Univariate	7
Rehfled (16)	2006a	Germany	SCLC	136	Protein	Reported	OS	Univariate	7
Rehfled (16)	2006b	Germany	NSCLC	91	Protein	Reported	OS	Univariate	6
Ren (17)	2017	China	CRC	118	Protein	Reported	OS	Multivariate	7
Shibata (18)	2002	Japan	ESCC	48	mRNA	Reported	OS	Univariate	7
Talvinen (19)	2009	Finland	BC	310	Protein	Reported	OS	Univariate	7
Wang (20)	2016	China	NSCLC	136	Protein	Reported	OS	Multivariate	7
Wei (21)	2015	China	RCC	192	Protein	Reported	OS	Multivariate	7
Wondergem (22)	2012	Singapore	RCC	179	mRNA	K-M curves	OS	Univariate	7
Zhang (23)	2014	China	ESCC	108	Protein	Reported	OS	Multivariate	7
Zhang (24)	2018	China	ESCC	76	Protein	Reported	OS	Univariate	6
Ma (25)	2020	China	LRGC	110	Protein	K-M curves	OS	Univariate	6
Demeure (26)	2013	America	ACC	22	Protein	Reported	OS	Univariate	6
Xu (27)	2016	China	GC	78	Protein	Reported	DFS	Multivariate	6
Cao (28)	2012	China	PC	64	Protein	Reported	DFS	Multivariate	6
Talvinen (30)	2008	Finland	BC	40	Protein	Reported	OS	Univariate	6
Ersvær (29)	2020	Norway	PC	243	Protein	Reported	RFS	Multivariate	6
Su (32)	2006	China	HCC	147	mRNA	K-M curves	OS	Univariate	7

HCC, hepatocellular carcinoma; RCC, Renal cell carcinoma; LUAD, Lung adenocarcinoma; ESCC, Eophageal squamous cell carcinoma; SCLC, Small cell lung cancer; NSCLC, Non-small cell lung cancer; MM, Multiple myeloma; CRC, Colorectal cancer; BC, Breast cancer; EC, Endometrial carcinoma; DTC, Differentiated thyroid carcinoma; PC, Prostate cancer; LRGC, Laryngeal cancer; GBM, Glioblastoma; OS, Overall survival; DFS, disease-free survival; RFS, recurrence-free survival.

### Association between high PTTG1 expression and OS

**Figure 2:**
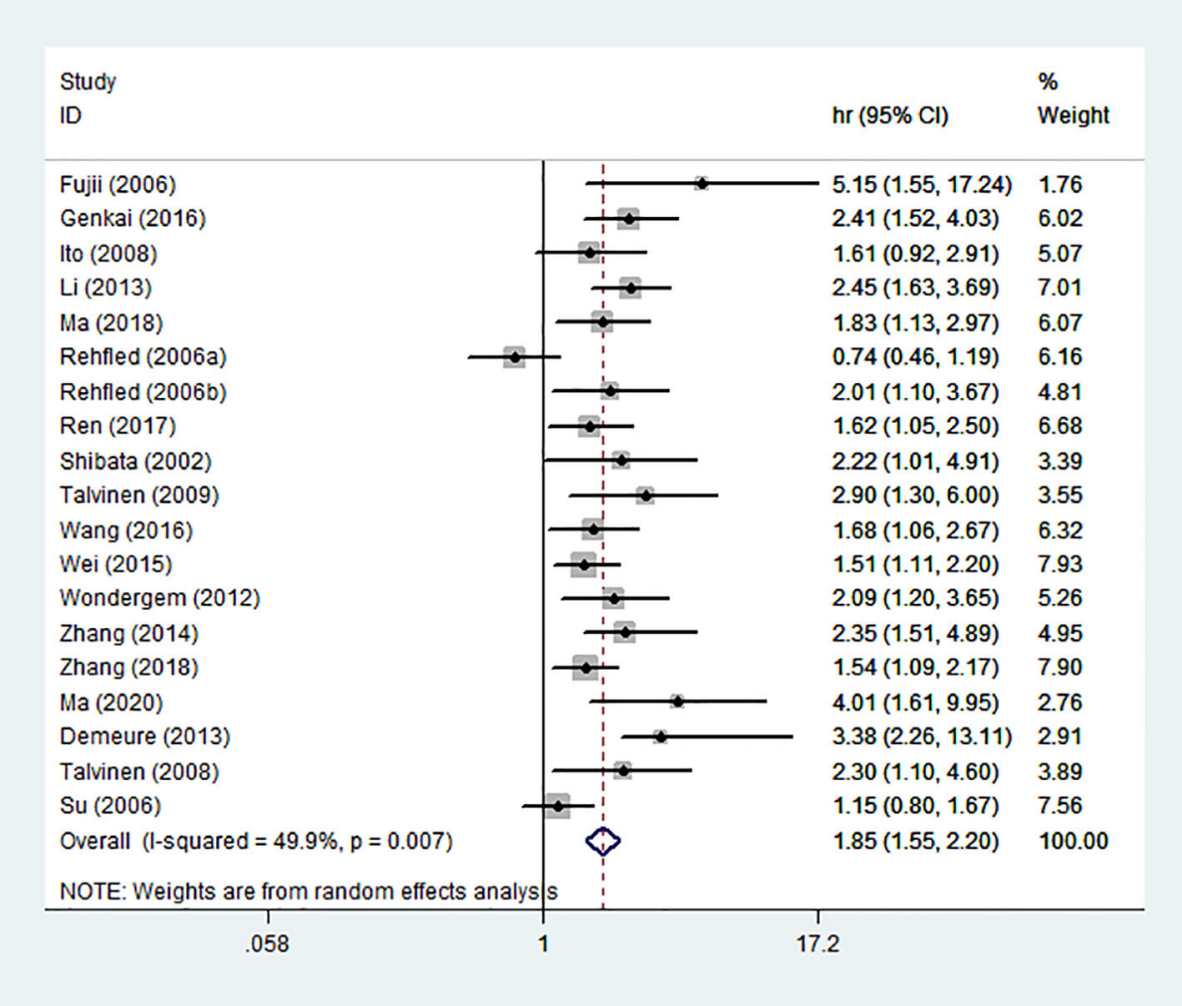
The forest plot for the correlation between high PTTG1 expression and OS.

19 studies reported OS data. Given the relatively low heterogeneity, we employed the fixed -effects model(I^2^ = 49.9). The pooled analysis demonstrated that elevated PTTG1 expression was significantly associated with worse OS, with a combined HR of 1.85 (95% CI: 1.55-2.20), indicating that patients with high PTTG1 expression had an approximately 85% increased risk of death compared with those with low expression (**Figure 2**). This consistent direction of effect across the majority of included studies suggested a robust adverse prognostic role of PTTG1.

### Subgroup analysis for OS

We conducted subgroup analysis by cancer type, sample size (≤100 vs >100), race(Caucasian vs Asian),analysis type (univariate vs multivariate), detection type (mRNA vs protein) and source of HR(reported vs KM) (**Table 2**). Subgroup analyses based on cancer type showed that high PTTG1 expression was mainly associated with poor OS(HR:1.65,95%CI:1.23-2.21),NSCLC (HR:2.06,95%CI:1.57-2.71),BC (HR: 2.56, 95%CI:1.52-4.32), ESCC (HR:1.74, 95%CI:1.35-2.23) and LRGC (HR:2.45, 95%CI:1.17-5.16), suggesting PTTG1 may serve as a universal prognostic biomarker for these tumors. Subgroup analysis based on detection method (protein vs mRNA) showed consistent associations between high PTTG1 expression and poor OS, suggesting that the prognostic value of PTTG1 was relatively stable across different detection approaches. The results of other subgroup analyses were consistent with the comprehensive results, which further confirmed the reliability of the combined results. Moderate heterogeneity was observed across studies. Subgroup analyses also suggested that cancer type,analysis type,sample size and race may sources of heterogeneity.

**Table 2:** Subgroup analysis for OS.

Variable	No of studies	Pooled HR (95%CI)	P-value	Heterogeneity		
				I^2^ (%)	P-value	Model
Cancer type						
HCC	2	2.17(0.51.27)	0.295	81.6	0.02	Random
RCC	2	1.65(1.23-2.21)	0.001	0	0.329	Fixed
NSCLC	3	2.06(1.57-2.71)	<0.01	0	0.485	fixed
BC	2	2.56(1.52-4.32)	<0.01	0	0.664	fixed
ESCC	4	1.74(1.35-2.23)	<0.01	0	0.589	fixed
LRGC	2	2.45(1.17-5.16)	0.018	55	0.136	Random
Others	4	1.68(0.92-3.09)	0.094	80.6	0.001	Random
Analysis type						
Univariate	13	1.91(1.50-2.43)	<0.01	61.2	0.002	Random
Multivariate	6	1.71(1.40-2.09)	<0.01	0	0.421	Fixed
Race						
Caucasian	5	1.92(1.04-3.53)	0.036	76.1	0.002	Random
Asian	14	1.77(1.56-2.01)	<0.01	30.5	0.133	Fixed
Detection type						
Protein	15	1.78(1.57-2.02)	<0.01	48.2	0.019	Fixed
mRNA	4	1.94(1.13-3.32)	0.016	63.6	0.041	Random
Sample size						
≥100	12	1.72(1.37-2.14)	<0.01	58.5	0.005	Random
<100	7	2.04(1.64-2.53)	<0.01	10.5	0.349	Fixed
Source of HR						
Reported	15	1.78(1.56-2.03)	<0.01	48.7	0.018	Fixed
KM	4	1.83(1.18-2.86)	0.007	63.3	0.042	Random

HCC, hepatocellular carcinoma; RCC, Renal cell carcinoma; NSCLC, Non-small cell lung cancer; BC, Breast cancer; ESCC, Eophageal squamous cell carcinoma; LRGC, Laryngeal cancer.

### Association between high PTTG1 expression and DFS/RFS

**Figure 3:**
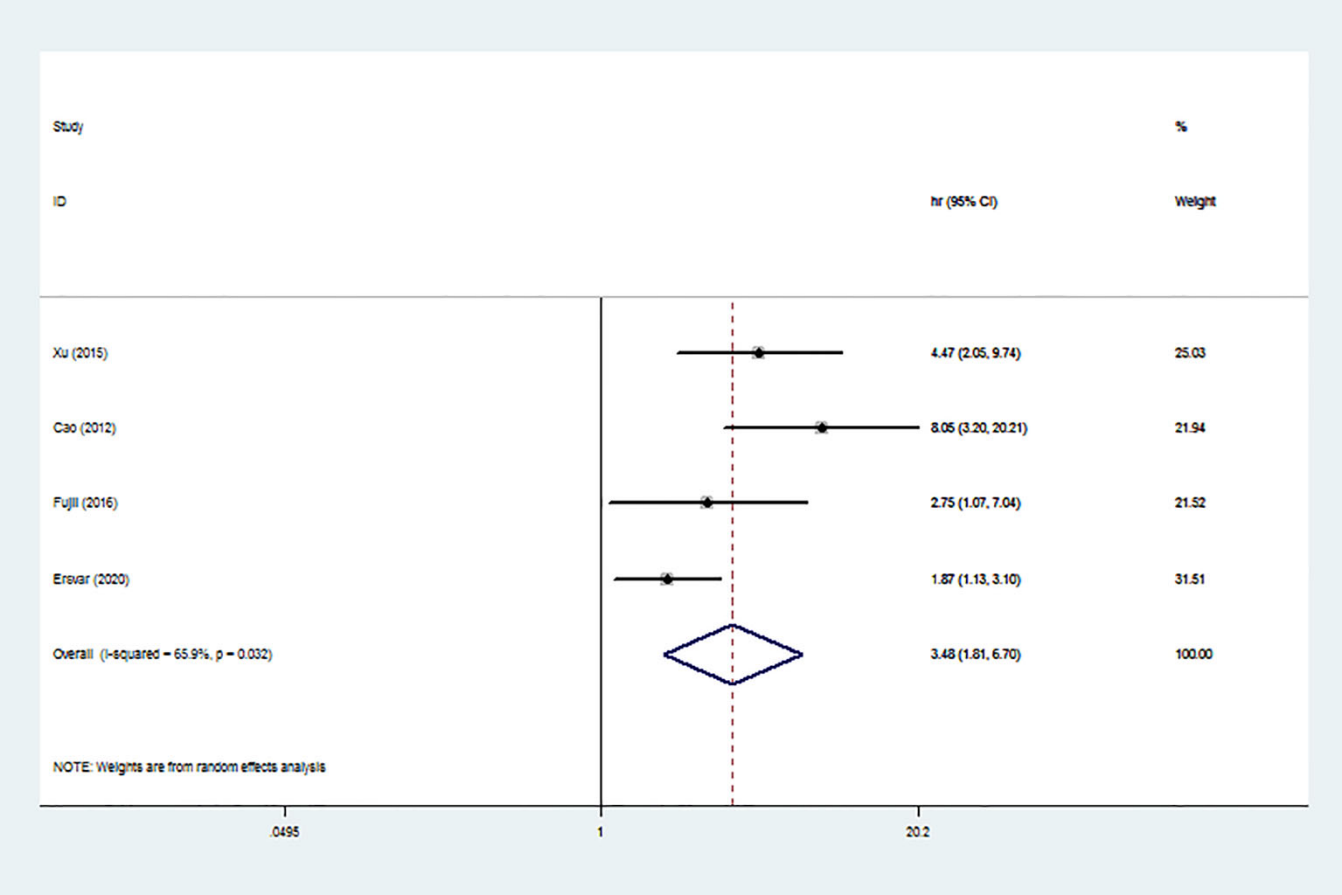
The forest plot for the correlation between high PTTG1 expression and DFS/RFS.

4 studies showed DFS/RFS data. We employed a random-effects model due to the obvious heterogeneity(I^2^ = 65.9). Elevated PTTG1 expression was also significantly associated with poorer DFS/RFS, with a pooled HR of 3.48 (95% CI: 1.81-6.70), corresponding to more than a threefold increased risk of recurrence or progression (**Figure 3**). The comparatively large effect size suggested that PTTG1 may be particularly relevant in early tumor progression and disease relapse. However, the limited number of studies warrants cautious interpretation of these findings.

### Association between high PTTG1 expression and clinicopathological features

Data about clinicopathological features was collected to further evaluate the association between high PTTG1 expression and clinicopathological features (**Table 3**). No significant association was observed between high PTTG1 expression and gender (Male vs Female) (OR:1.24, 95% CI: 0.94-1.63) and tumor differentiation (Poor vs Moderate/Well)(OR:1.54, 95% CI: 0.51-4.60). However, high PTTG1 expression was significantly associated with tumor stage (III-IV vs I-II) (OR:3.92, 95% CI: 2.97-5.18), T stage (T3–4 vs T1-2) (OR:2.47, 95% CI: 1.33-4.59), lymphatic invasion (Yes vs No) (OR:3.39, 95% CI: 1.78-6.46) and metastasis (Yes vs No) (OR 7.47, 95% CI:2.82-19.81). Notably, the strongest association was observed for metastasis, suggesting that PTTG1 may play a particularly important role in tumor dissemination and invasive progression. These findings indicate that elevated PTTG1 expression was strongly linked to tumor aggressiveness, invasion, and metastatic potential, supporting its biological role in cancer progression rather than merely reflecting prognosis.

**Table 3:** Correlation between PTTG1 expression and clinicopathologic characteristics in solid tumors.

Variable	No. of studies	Estimate OR (95%CI)	*p*-value	Heterogeneity	
				I^2^(%)	*p*-value
Gender (Male VS Female)	10	1.24(0.94-1.63)	0.14	21.4	0.25
Tumor stage (III-IV VS I-II)	12	3.92(2.97-5.18)	<0.01	25	0.2
T stage (T3–4 VS T1-2)	5	2.47(1.33-4.59)	<0.01	56.2	0.06
Lymphatic invasion (N+ VS N-)	11	3.39(1.78-6.46)	<0.01	69.4	<0.01
Metastasis (M+ VS M-)	7	7.47(2.82-19.81)	<0.01	59.3	0.02
Tumor differentiation	6	1.54(0.51-4.60)	0.44	86.4	<0.01
(Poor VS Moderate/Well)					

### Sensitivity analysis

**Figure 4:**
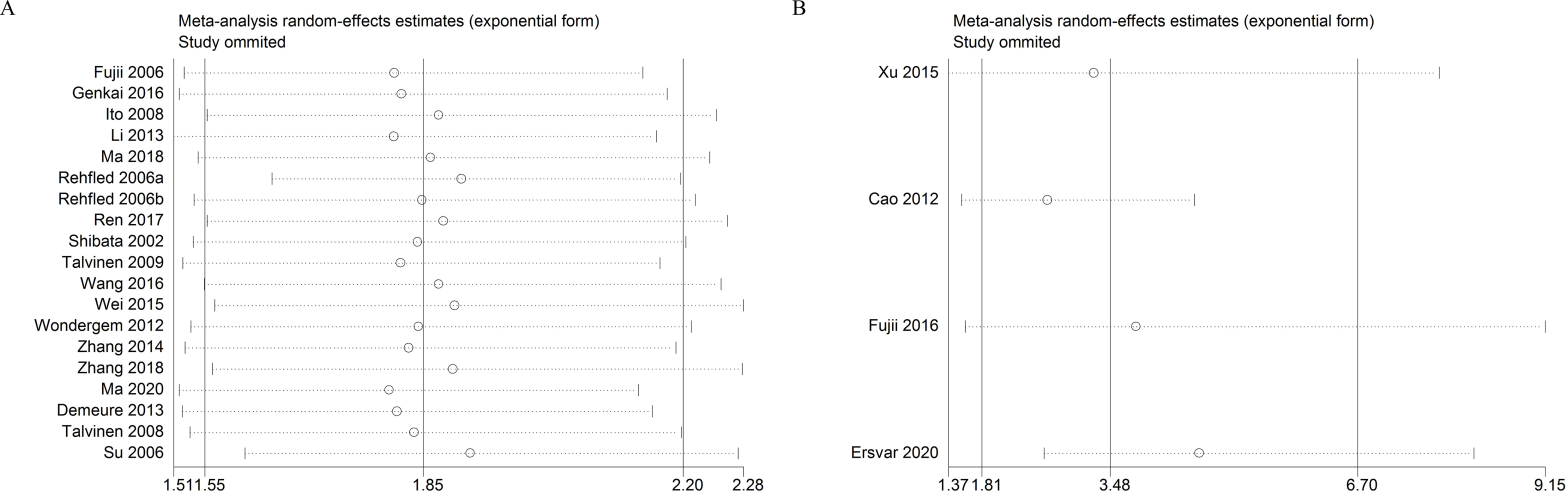
Sensitive analysis. **(A)** Sensitive analysis for OS. **(B)** Sensitive analysis for DFS/RFS.

To evaluate sensitivity, we sequentially excluded each individual study to explore their influence on the pooled OS and DFS/RFS (**Figures 4A, B**). The pooled HRs were stable, indicating that none of the included studies largely affected the results.

### Publication bias

**Figure 5:**
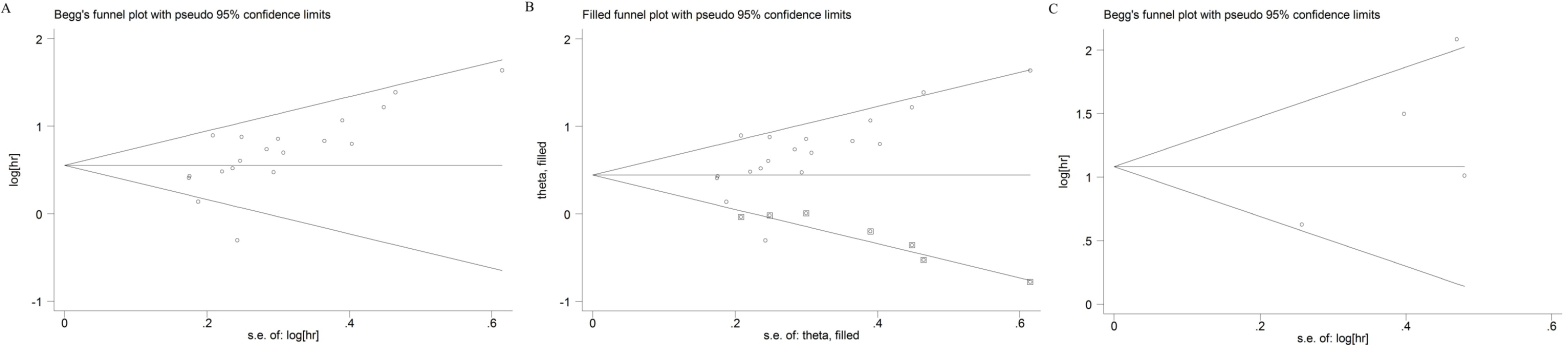
Publication bias. **(A)** Publication bias for OS. **(B)** trim-and-fill method for OS. **(C)** Publication bias for DFS/RFS.

We further evaluated the publication bias. The funnel plot for OS was asymmetric.The P values of Begg’s test and Egger’s test were 0.001 and 0.004, respectively (**Figure 5A**). Then, we utilized the trim-and-fill method to correct the funnel asymmetry and recalculate the estimated HRs. The corrected funnel plot became symmetrical and the recalculated HR was 1.559 (95% CI: 1.305-1.861), suggesting that the results of the meta-analysis were stable (**Figure 5B**). For DFS/PFS, the funnel plot showed a slight degree of asymmetry, which may indicate potential small-study effects or underlying heterogeneity among studies (**Figure 5C**). However, Begg’s test(P = 0.734) and Egger’s test (P = 0.197) did not demonstrate statistically significant publication bias.

## Discussion

To the best of our knowledge, this study was the first meta-analysis to comprehensively assess the prognostic value and possible mechanisms of PTTG1 expression in various cancers. In the study, a total of 22 studies from 21 articles enrolling 2673 patients were included. We found that higher PTTG1 expression was significantly associated with shorter OS and DFS/RFS. Subgroup analysis showed that PTTG1 may be a more suitable prognostic marker for RCC,NSCLC, BC, ESCC and LRCC. Importantly, the prognostic effect size observed in the present meta-analysis appears to be clinically meaningful rather than merely statistically significant. The pooled HR for OS indicated that elevated PTTG1 expression was associated with an approximately 85% increased risk of mortality, which represents a moderate-to-strong prognostic effect size in the context of oncology biomarker studies. Moreover, the pooled HR for DFS/RFS exceeded 3.0, suggesting that PTTG1 overexpression may be particularly relevant to early recurrence, tumor dissemination, and disease progression. Notably, the prognostic impact of PTTG1 was accompanied by strong associations with aggressive clinicopathological features, especially distant metastasis and advanced tumor stage. These relatively large effect sizes supported the biological plausibility that PTTG1 was not simply a passive prognostic marker, but may actively participate in tumor progression and metastatic behavior.

The observed heterogeneity across studies was likely multifactorial and warrants cautious interpretation. Firstly, biological heterogeneity among tumor types may substantially influence the prognostic value of PTTG1. Although elevated PTTG1 expression was consistently associated with adverse outcomes in several malignancies, including RCC, NSCLC, ESCC, BC, and LRGC, the magnitude of prognostic impact varied considerably, suggesting tumor-specific biological functions and signaling interactions. Secondly, methodological variability may also contribute to heterogeneity. Different studies employed distinct assay platforms. Protein-based and mRNA-based measurements may not fully reflect equivalent biological states because post-transcriptional regulation and subcellular localization can significantly influence PTTG1 activity. In addition, protein expression detected by immunohistochemistry is susceptible to variability in antibody specificity, staining procedures, scoring systems, and cut-off values, whereas mRNA expression analyses may differ in normalization strategies and analytical pipelines. These methodological inconsistencies may reduce inter-study comparability and influence the pooled effect estimates. Thirdly, the absence of standardized cut-off definitions represents another important source of heterogeneity. Various studies used median values, staining scores, percentage positivity, or arbitrary thresholds to define high PTTG1 expression, thereby limiting reproducibility across studies. Finally, clinical heterogeneity, including differences in treatment regimens and disease stage distributions, may further modify the prognostic relevance of PTTG1. Collectively, these findings suggest that the prognostic value of PTTG1 should be interpreted within specific biological and clinical contexts rather than considered universally equivalent across all solid tumors. Future studies should establish standardized detection methods and unified cut-off criteria to improve reproducibility and clinical applicability.

An important yet underrecognized issue in interpreting PTTG1 as a prognostic biomarker is that its clinical significance may depend not only on overall expression levels but also on subcellular localization. Biologically, this distinction is meaningful because nuclear PTTG1 is generally considered the transcriptionally active form involved in gene regulation, chromosomal instability, and tumor progression, whereas cytoplasmic PTTG1 may reflect relatively inactive or storage-associated states. Mechanistically, nuclear translocation of PTTG1 has been linked to tumor aggressiveness. PTTG1-binding factor (PBF) facilitates the nuclear import of PTTG1 and enhances its oncogenic activity, thereby promoting tumor proliferation and invasion (37, 38). In addition, nuclear PTTG1 may interact with transcriptional regulators such as ZEB1, contributing to epithelial-mesenchymal transition (EMT) and metastatic progression (39). Interactions with proteins including SPTBN1 further suggest potential roles in cytoskeletal regulation and genome stability (40). Collectively, these findings indicate that nuclear localization may represent a biologically active state of PTTG1 associated with more aggressive tumor behavior. From a clinical perspective, assessment of total PTTG1 expression alone may be insufficient to fully capture its prognostic relevance. Conventional approaches, including bulk-tissue mRNA quantification and non-compartmentalized immunohistochemical scoring, did not distinguish nuclear from cytoplasmic localization and may therefore obscure clinically meaningful differences. In the present meta-analysis, the included studies assessed overall PTTG1 expression without stratifying subcellular localization, which may partly explain the observed heterogeneity. Future studies should therefore incorporate standardized evaluation of nuclear and cytoplasmic PTTG1 distribution and validate the independent prognostic value of nuclear PTTG1 in prospective cohorts.

The mechanisms underlying the oncogenic role of PTTG1 in tumors are complex and multifaceted. Accumulating evidence suggests that PTTG1 promotes tumor initiation, proliferation, invasion, metastasis, and resistance to anticancer therapy through the regulation of multiple signaling pathways and cellular processes. Cho-Rok et al. demonstrated that PTTG1 overexpression significantly enhanced hepatocellular carcinoma growth both in vitro and in vivo by negatively regulating p53-induced apoptosis (41). In cholangiocarcinoma, Hu et al. reported that PTTG1 activated the MAPK signaling pathway, thereby promoting tumor cell proliferation and suppressing apoptosis (42). In prostate cancer, PTTG1 was markedly upregulated in both tumor tissues and cell lines, while its knockdown inhibited cancer cell proliferation through G1-phase cell cycle arrest (43). Moreover, PTTG1 has been shown to promote prostate cancer progression by downregulating mothers against decapentaplegic homolog 3 and functioning as a downstream target of the androgen receptor pathway (44, 45). In lung adenocarcinoma, high PTTG1 expression was associated with tumor metastasis, and silencing PTTG1 suppressed tumor growth and invasion via activation of the TGF-β1/SMAD3 signaling pathway (46). Similarly, Xie et al. demonstrated that PTTG1 promoted breast cancer cell proliferation through nuclear exclusion of p27, while additional studies indicated that elevated PTTG1 expression facilitated epithelial–mesenchymal transition (EMT) and expansion of the breast cancer stem cell population (47). In glioma, downregulation of PTTG1 induced apoptosis and inhibited tumor cell proliferation (48). In ESCC, PTTG1 promoted EMT through activation of glioma-associated oncogene homolog 1 expression (9). Furthermore, PTTG1 overexpression enhanced thyroid cancer cell proliferation by upregulating vascular endothelial growth factor and VEGF receptor type 1 and activating the MAPK pathway (49). Emerging evidence also indicates that PTTG1 participates in multiple oncogenic signaling networks. Upregulation of PTTG1 contributed to JAK2V617F/STAT5 signaling-induced proliferation in HEL cells (50). In colorectal cancer, PTTG1 acted as a downstream target of STAT3, and its suppression significantly inhibited tumor growth and colony formation both in vitro and in vivo (51). In seminoma, PTTG1 overexpression promoted tumor migration and invasion through activation of MMP-2 and ZEB1 (39, 52, 53). Additionally, increasing evidence suggests that PTTG1 is involved in microRNA- and long non-coding RNA-mediated regulation of proliferation and apoptosis in multiple malignancies, including cervical cancer and gastric cancer (54, 55). Collectively, these findings indicate that PTTG1 functions predominantly as an oncogenic driver across a wide range of solid tumors and hematological malignancies.

Several shortcomings in the study should be seriously considered. Firstly, survival data in 4 studies was obtained indirectly from Kaplan-Meier survival curves, which may inevitably introduce estimation bias. Secondly, most of included studies were from Asia, so the results of this study may be more representative of Asian populations. Thirdly, the number of studies exploring the prognostic value of PTTG1 in each different type of cancers was limited. Therefore, more studies were required to further determine the association of PTTG1 with prognosis in each specific cancer. Fourthly, all the included studies only detected PTTG1 expression in tissues, and the relationship between serum PTTG1 expression and cancer prognosis should further be explored. Fifthly, the limited number of studies reporting DFS/RFS outcomes precluded detailed cancer-specific analyses, which may affect the generalizability of these findings across different tumor types. Sixthly, the majority of studies included in this meta-analysis did not distinguish between nuclear and cytoplasmic PTTG1 expression, which may obscure its true prognostic significance. Finally, due to the lack of complete data, we could not determine which time point or stage the PTTG1 expression in cancers performs best as a prognostic marker.

Several strengths should be highlighted. Firstly, this study provides a comprehensive and quantitative evaluation of the prognostic value of PTTG1 across multiple solid tumors, based on a relatively large pooled sample size. Compared with individual studies, this approach improved statistical power and enhanced the reliability of the conclusions. Secondly, the robustness of the findings was supported by multiple analytical strategies. Sensitivity analyses demonstrated that the pooled results were stable and not driven by any single study. In addition, publication bias was rigorously assessed using Begg’s test and Egger’s test, and further corrected using the trim-and-fill method, with consistent results observed after adjustment. Thirdly, this study not only evaluated survival outcomes but also systematically analyzed clinicopathological parameters, including tumor stage, lymphatic invasion, and metastasis. This integrative approach strengthened the biological plausibility of the findings and provided a more comprehensive understanding of the role of PTTG1 in tumor progression. Fourthly, subgroup analyses and meta-regression were performed to explore potential sources of heterogeneity. These analyses identified tumor type as a key contributor, thereby improving the interpretability of the results and highlighting context-specific effects. Finally, this study emphasized the clinical relevance of PTTG1 by linking its expression to meaningful clinical outcomes and aggressive tumor features.

In conclusion, the meta-analysis indicated that high PTTG1 expression was significantly associated with unfavorable survival outcomes and aggressive clinicopathological characteristics in solid tumors, suggesting that PTTG1 may serve as a promising prognostic biomarker and potential therapeutic target. Patients with elevated PTTG1 expression could represent a high-risk subgroup with increased likelihood of disease progression, recurrence, and mortality. This information may support more individualized management strategies. However, due to several inherent limitations, its clinical implementation required further validation through large-scale prospective studies. Standardization of detection methods and cut-off definitions is needed in future studies to improve the clinical applicability of PTTG1 as a prognostic biomarker.

## Data Availability

The original contributions presented in the study are included in the article/**Supplementary Material**. Further inquiries can be directed to the corresponding author.
